# MOF-808/graphene oxide composite as an efficient adsorbent for urea removal: experimental and atomistic study

**DOI:** 10.1038/s41598-026-51802-6

**Published:** 2026-05-04

**Authors:** Nahid Khandan, Meysam Habibi, Reza Maleki

**Affiliations:** 1 Department of Chemical Technologies, Iranian Research Organization for Science and Technology (IROST), P.O. Box 33535111, Tehran, 3313193685 Iran; 2 AGAP2 Engineering Consultant Company, Strada 1 Palazzo CC, 20057 Assago, Milan Italy

**Keywords:** MOF, Graphene oxide composite, Urea removal, Artificial kidneys, Adsorption mechanism, Experimental methods, Atomistic study, Chemistry, Materials science, Nanoscience and technology

## Abstract

This study explores the synthesis and evaluation of a novel composite material based on the in-situ growth of Metal-Organic Framework (MOF) on graphene oxide composite for the removal of urea from aqueous environments, particularly for artificial kidney applications. The synthesized MOF-808@rGO composite exhibits enhanced stability, tailored surface properties, and tunable pore structures, enabling effective urea adsorption. Both experimental methods and atomistic simulations were employed to investigate the interaction mechanisms between urea molecules and the composite. The synthesis process involves the in-situ growth of MOF-808 on GO surfaces, followed by heat treatment to produce the MOF-808@rGO composite. The resulting material was characterized using BET, SEM, PXRD, and FTIR analyses. Performance results indicate that the composite can achieve up to 755 mg/g of urea adsorption at a concentration of 2000 ppm, showing excellent recyclability over multiple adsorption cycles. The results demonstrate a significant improvement in urea adsorption capacity compared to the individual components. This enhancement is attributed to the synergistic effects of MOF-808 porosity and the presence of graphene oxide, which improve MOF dispersion, interfacial charge distribution, and the accessibility of adsorption sites. In-situ growth of MOF-808 on graphene oxide enhances pore accessibility and modifies interfacial surface chemistry, leading to increased urea–adsorbent interactions and improved structural stability of the composite. Notably, the synthesized composite maintained structural integrity and demonstrated reusability, addressing challenges related to the regeneration of adsorbents in clinical settings. The performance metrics were evaluated under simulated dialysis conditions, revealing promising outcomes for integrating these materials into portable dialysis systems. The findings contribute valuable insights to the optimization of MOF-graphene composites, paving the way for next-generation solutions in kidney disease management. This work underscores the potential of the MOF-808/graphene oxide composite as an innovative approach to enhance urea removal efficiency, ultimately benefiting patients with compromised renal function and improving the efficacy of dialysis technologies.

## Introduction

MOFs combined with graphene-based materials have emerged as promising adsorbents for efficient urea removal in artificial kidney applications, owing to their enhanced stability and suppressed component aggregation^1,2^. Their tunable porosity and surface chemistry enable effective urea adsorption, making them attractive candidates for improving dialysis performance^3,4^. Recent efforts have focused on optimizing MOF structures to enhance urea selectivity and adsorption capacity, which are critical for renal replacement therapies^5,6^. Although transition-metal-based MOFs are widely explored, MOFs derived from s-block elements offer advantages such as lower toxicity and cost, particularly for biomedical applications^7^. Nevertheless, commonly reported urea-adsorbing MOFs, including UiO-66-NH₂ and Zn-ZIF, exhibit limited chemical stability under dialysis conditions, which restricts their reusability. Incorporating graphene into MOF-derived composites offers a viable strategy to overcome these limitations^8^ by improving structural robustness and adsorption performance^9,10^. Graphene possesses outstanding mechanical and electrical properties that make it an attractive component in adsorption-based composites^11,12^; however, restacking of graphene layers driven by π–π interactions can significantly reduce its accessible surface area and functional performance. Forming composites with complementary materials, such as MOFs, has therefore been proposed to mitigate aggregation and enhance adsorption efficiency^13,14^. In particular, few-layer graphene (FLG) enables better control over surface accessibility and interfacial interactions when integrated into porous frameworks^15,16^. The incorporation of MOF-derived materials onto graphene improves structural stability, surface area, and mass transport, resulting in enhanced adsorption performance for urea and related applications^17,18^. Despite progress in adsorbent development, achieving high efficiency, selectivity, and long-term stability under physiologically relevant conditions remains challenging^19,20^. Addressing these limitations is especially critical in renal replacement technologies, where large daily urea loads must be removed to maintain metabolic homeostasis^6,21^. In this context, graphene/MOF-derived composites offer a promising platform that combines the robustness and conductivity of graphene with the tunable adsorption characteristics of MOFs, enabling improved urea management in artificial kidney systems^2,8,12,22^. Urea is the primary nitrogenous waste in the human body, with a daily production rate of approximately 240–470 mmol. Its accumulation above ~ 20 mM is toxic, necessitating efficient removal in patients with renal failure. In portable and wearable artificial kidney systems, urea removal remains particularly challenging due to its high solubility, low chemical reactivity, and large daily load. Conventional approaches, such as enzymatic hydrolysis and electrochemical oxidation, are limited by by-product formation, system complexity, and poor operational stability, restricting their applicability in compact dialysis devices. Consequently, adsorption-based strategies have attracted increasing attention as viable alternatives for next-generation dialysis technologies^5,11^. An effective urea adsorbent must combine high capacity, rapid kinetics, aqueous stability, and reusability while maintaining minimal weight and volume^5,11^. However, traditional materials including activated carbon, zeolites, and polymeric resins generally exhibit insufficient urea affinity or require impractically large quantities^11,22,23^. This limitation motivates the development of advanced porous adsorbents with tailored surface chemistry^5,24–26^. Recent advances in synthesis strategies have enabled graphene/MOF composites with well-defined pore architectures, resulting in enhanced urea adsorption compared to conventional materials^26–28^. Incorporating urea during synthesis promotes metal-ion intercalation within graphene layers, facilitating exfoliation under mild conditions and enabling fine control over conductivity and rheological properties^8,22^. Carbonization of MOF precursors further increases surface area and porosity, yielding structures highly favorable for adsorption applications^27,29^. For instance, MOF-5-derived carbons can achieve BET surface areas exceeding 1500 m^2^/g, which may be increased to nearly 3000 m^2^/g through precursor modification^1,29^. Graphene, particularly when doped with heteroatoms such as nitrogen or phosphorus, exhibits enhanced urea affinity through polar interactions and hydrogen bonding^30^. Studies have shown that nitrogen-doped graphene nanosheets exhibit remarkable adsorption performance, significantly enhancing affinity for urea removal^31^. The synergistic combination of porous MOF-derived frameworks and graphene-based components significantly improves urea uptake relative to traditional adsorbents^18^. Moreover, MOF pore size, surface chemistry, and hydrophilicity can be tailored during or after synthesis to optimize urea–adsorbent interactions^11,23^. Compared with enzymatic and electrochemical urea removal methods, which are costly and operationally complex^32,33^, graphene/MOF composites offer a scalable, efficient, and potentially lower-cost alternative for artificial kidney applications^34–37^.

Recent studies increasingly employ molecular and atomistic simulations to elucidate urea–adsorbent interactions and guide the rational design of materials with improved removal efficiency^38,39^. MOFs have demonstrated considerable potential in biomedical applications, particularly for the adsorption and conversion of uremic toxins. Li-Er Deng et al.^40^ reported the effectiveness of MOFs as adsorbents for uremic species^40^. Hua Yu et al.^41^ showed that nickel–cobalt MOFs enable efficient urea electrooxidation under simulated dialysis conditions^41^. Similarly, Jie Ren et al.^42^ demonstrated photo-electrolysis of urea using nickel-based MOFs^42^. Beyond removal, MOF-based composites have been explored for urea detection. Cancan Bao et al.^43^ developed nickel-MOF non-enzymatic sensors with high sensitivity and selectivity in biological fluids^43^. Complementary computational screening by Fabiani et al.^44^ identified nanoporous materials, including MOFs, with high urea selectivity in aqueous environments relevant to dialysis processes^44^.

Most reported MOF–carbon composites employ carbonaceous supports primarily to enhance conductivity or surface area, often relying on weak physical adsorption for polar molecules such as urea. In contrast, the MOF-808@rGO composite offers distinct structural and chemical advantages. Upon activation, MOF-808 exposes unsaturated Zr(IV) sites that act as strong Lewis acid centers, enabling specific interactions with urea through carbonyl coordination and hydrogen bonding. Reduced graphene oxide provides a 2D scaffold that improves MOF dispersion, suppresses particle agglomeration, and increases accessible adsorption sites. This synergistic integration promotes combined chemisorption–physisorption mechanisms, as supported by experimental adsorption results and atomistic simulations^45–50^. MOF-808 was selected due to its unique combination of structural and chemical features that favor urea adsorption. Its Zr(IV) nodes provide abundant Lewis acidic sites that selectively interact with urea’s polar amide groups. The framework offers large, hydrophilic pores that facilitate urea diffusion and retention while maintaining structural integrity under aqueous conditions. When integrated with rGO, strong interfacial interactions enhance stability, electron density, and adsorption kinetics. Furthermore, the well-defined Zr_6_ clusters enable atomistic simulations, allowing direct correlation between experimental data and molecular-level insights^48–50^.

This study integrates MOF-808 and graphene, leveraging their complementary advantages to enhance structural stability and urea adsorption efficiency. For the first time, this composite is investigated using both experimental and simulation approaches. The results demonstrate superior adsorption capacity compared with previously reported materials. They also introduce a durable and reusable adsorbent suitable for dialysis applications, addressing a gap in existing research on graphene oxide/MOF-808 composites for urea removal.

## Materials and methods

The MOF-808@rGO composite was synthesized via an in-situ solvothermal method. This method is widely used for the in-situ growth of MOF-808 on graphene oxide, as it facilitates reactions under elevated temperature and pressure to promote crystallization^38,39^. Unlike hydrothermal methods, which employ aqueous media, solvothermal synthesis utilizes organic solvents^22,38^. The synthesis generally includes preparation of a precursor solution, controlled reaction conditions to facilitate crystal growth, followed by separation, washing, and drying. Additional thermal treatment and vacuum drying are typically applied to enhance material properties and ensure purity of the final composite^7,51^.

### Synthesis of MOF-808@rGO

**Fig. 1:**
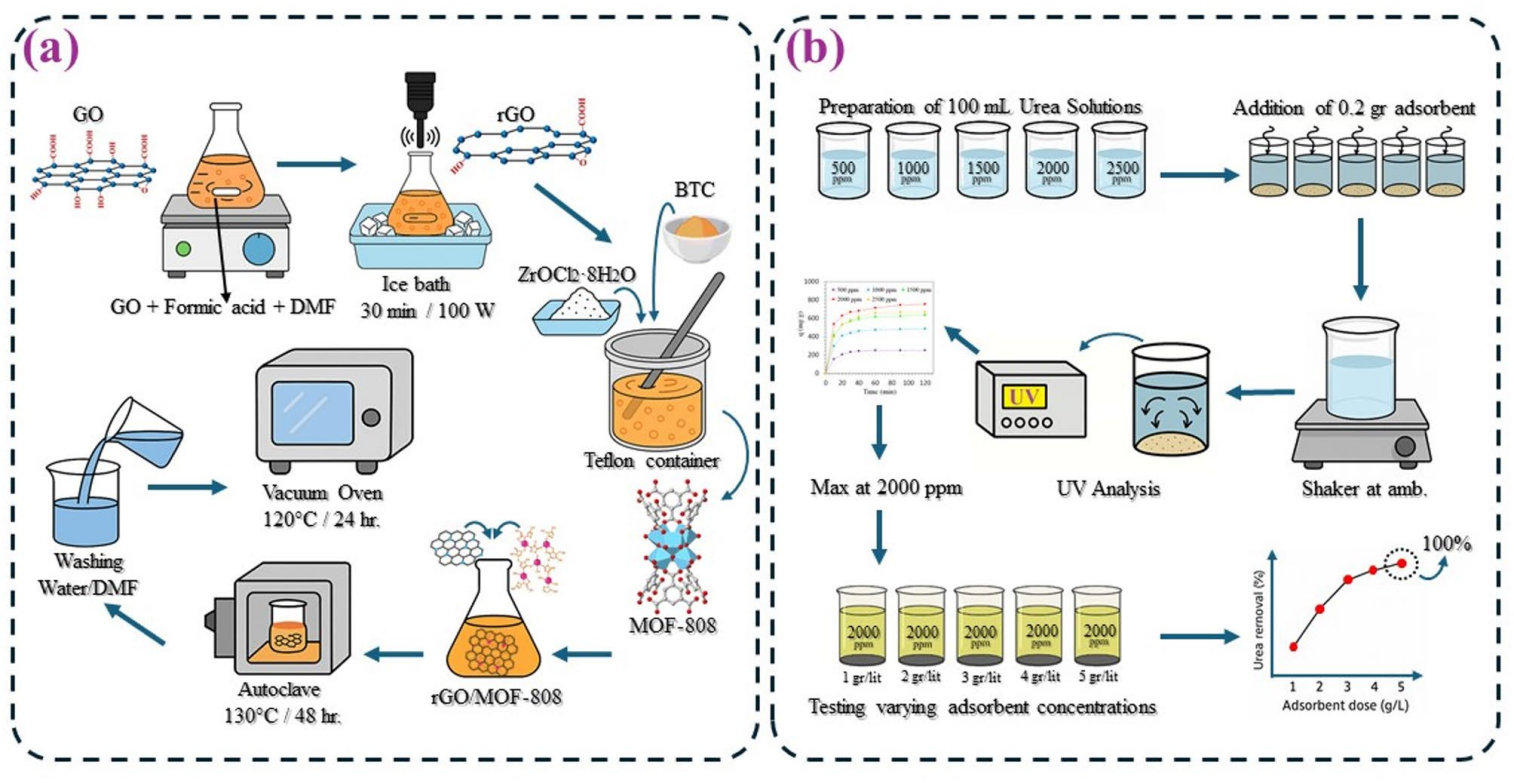
(**a**) Schematic MOF-808@rGO composite synthesis, highlighting the dispersion of graphene oxide in solvents followed by thermal treatment to integrate with MOF-808, depicting zirconium clusters coordinated with BTC linkers. (**b**) Effect of adsorbent dosage on urea removal efficiency. Nearly 100% of the urea in the 2000 ppm solution was removed when the adsorbent dose was increased to 5 g/L.

As illustrated in Fig. 1a, the following processes are employed in the synthesis:

Graphene oxide (100 mg) was dispersed in a mixed solvent of formic acid (22.5 mL) and dimethylformamide (DMF, 22.5 mL). The mixture was magnetically stirred for 30 min, followed by ultrasonication in an ice bath for 30 min at 100 W (30s pulse/rest cycles) to obtain a homogeneous and stable suspension.

Following GO dispersion, zirconyl chloride octahydrate (ZrOCl_2_·8H_2_O, 0.485 g) and benzene-1,3,5-tricarboxylic acid (BTC, 105 mg) were added to the suspension in a Teflon-lined container. The mixture was magnetically stirred for 1 h to ensure complete dissolution and homogeneous mixing. The metal precursor and organic linker subsequently interacted within the GO dispersion, enabling the in-situ formation of the MOF-808 framework. This process facilitated the uniform growth of MOF-808 onto the graphene oxide surface, leading to the formation of the MOF-808@rGO composite.

The resulting mixture was transferred into a 250 mL Teflon-lined autoclave and heated at 130 °C for 48 h. After cooling to room temperature, the product was collected by filtration, yielding a dark gray solid. This solvothermal treatment enabled the formation of the MOF-808 framework and its integration with the graphene-based support, resulting in the MOF-808@rGO composite.

The obtained product was washed several times with water and DMF to remove residual reactants and impurities. This dual washing approach guarantees a high-purity MOF-808@rGO composite with optimal properties. It was then subjected to solvent exchange by sequential immersion in DMF, water, and acetone for 3 days each, with periodic solvent replacement. Finally, the material was dried under vacuum at 100 °C for 24 h to yield the activated MOF-808@rGO composite^52–54^.

### Performance evaluation of MOF-808@rGO for urea removal

To evaluate the performance of the adsorbent, five 100 mL urea solutions with concentrations of 500, 1000, 1500, 2000, and 2500 ppm were prepared in separate beakers. Then, 0.2 g of adsorbent was added to each solution, and the mixtures were placed in a shaker at ambient temperature under moderate stirring.

The percentage of urea removed was determined using UV analysis by tracking changes in urea concentration over time. This technique enables evaluation of the adsorption behavior, rate, and efficiency of MOF-808@rGO, and provides a rapid, non-destructive, and reliable method for real-time monitoring^55,56^.

The maximum adsorption capacity of the adsorbent was approximately 755 mg/g at an initial urea concentration of 2000 ppm. Based on this result, the adsorbent dosage was varied (1–5 g/L) in additional experiments to achieve complete urea removal at this concentration. As shown in Fig. 1b, increasing the adsorbent dose to 5 g/L resulted in nearly 100% urea removal from the 2000ppm solution.

At the atomic level, adsorption is governed by physical and chemical interactions between urea molecules and the MOF-808@rGO adsorbent. The process follows a dynamic equilibrium, in which urea molecules bind to the adsorbent surface until all available active sites are occupied. These active sites enable the binding of urea molecules, and increasing the adsorbent dosage enhances the total available surface area. At lower adsorbent doses (1–4 g/L), the number of available adsorption sites is insufficient, and some urea molecules remain unbound, resulting in incomplete removal. In contrast, at an adsorbent dose of 5 g/L, sufficient active sites are available to accommodate all urea molecules, leading to complete removal.

## Results and discussions

The development of wearable dialysis systems requires compact, lightweight components, particularly for the dialysis fluid regeneration unit. Surface adsorption offers an effective strategy for urea removal, as it avoids chemical decomposition and the formation of secondary by-products, eliminating the need for additional purification steps. Therefore, adsorbents with high capacity, stability in aqueous environments, and reusability are essential.

In this study, MOF-808 was selected due to its stability in water and adsorption potential; however, its relatively large pore size compared to MOFs such as ZIF-based materials may limit efficiency for small molecules like urea. To address this limitation, a MOF-808/graphene oxide composite was developed to tune pore structure and enhance adsorption performance.

The resulting composite exhibited improved urea adsorption capacity compared with individual components, along with good aqueous stability and recyclability. Structural modification led to reduced and optimized pore dimensions, promoting stronger urea confinement within the framework and improving overall adsorption efficiency. These findings highlight the importance of pore engineering in enhancing MOF-based adsorbents for wearable dialysis applications.

**Fig. 2:**
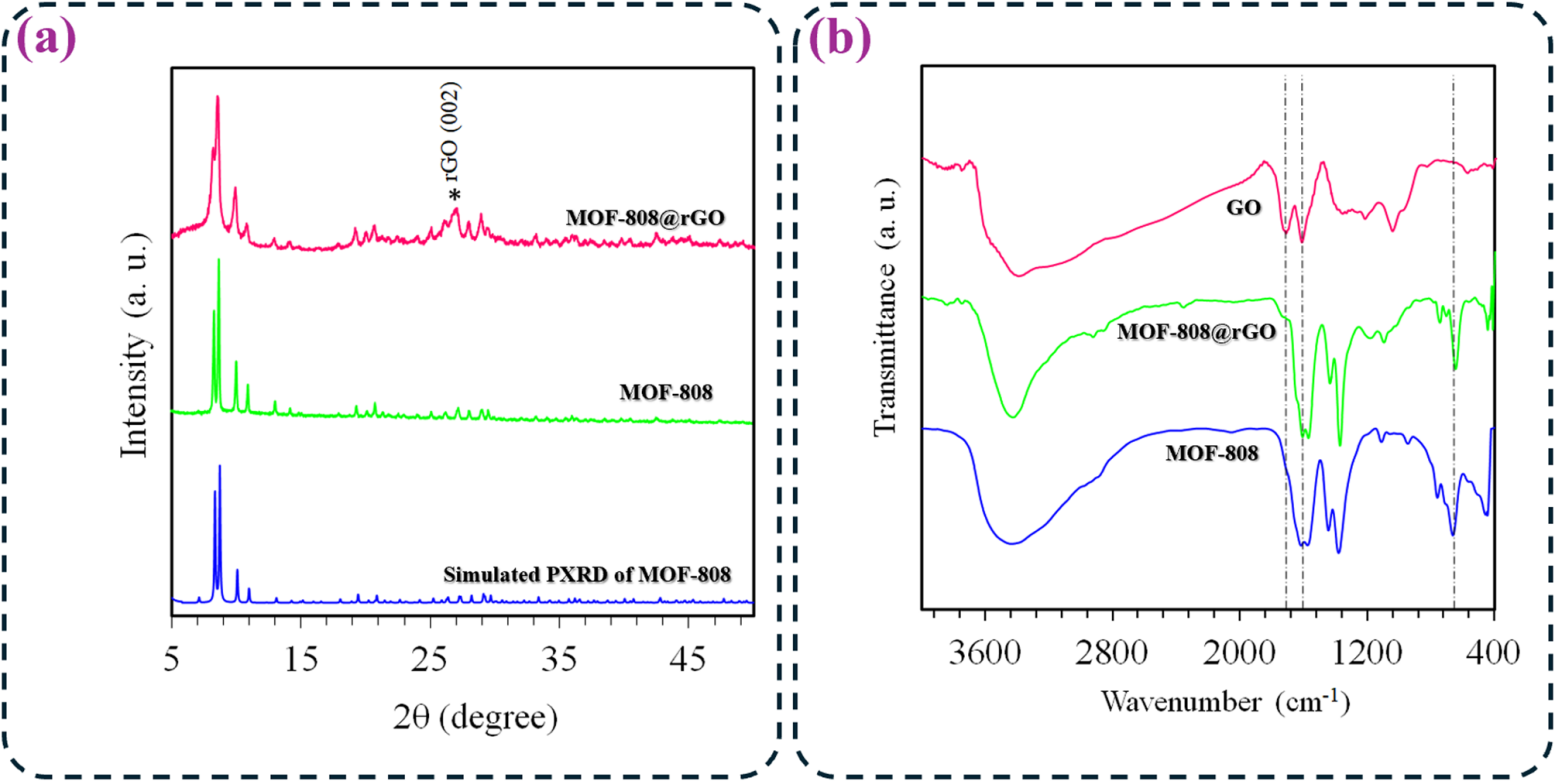
(**a**) XRD patterns of the simulated pattern based on single-crystal X-ray (PXRD) data of MOF-808, MOF-808, and MOF-808@rGO. (**b**) FT-IR Spectra of the MOF-808, MOF-808@rGO, and GO.

Figure 2a depicts the XRD patterns of three samples, providing insight into their structural evolution during synthesis. The simulated MOF-808 pattern, derived from single-crystal data, exhibits sharp and well-defined peaks, reflecting ideal crystallinity and serving as a reference. The synthesized MOF-808 shows similar diffraction peaks, although slightly broadened or shifted, confirming successful formation of the framework. Peak broadening may indicate reduced crystallite size or minor structural disorder. In the composite sample (Fig. 2a), the characteristic GO peak at ~ 10°, associated with large interlayer spacing, disappears. Instead, a broad peak appears around 25–27°, corresponding to the (002) plane of rGO. This shift indicates the removal of oxygen-containing groups and intercalated water, leading to restacking of graphene layers with reduced interlayer spacing^57^. These changes confirm the reduction of GO to rGO during in-situ composite formation. The (002) peak is characteristic of layered graphene structures, while MOF-808 peaks may exhibit slight suppression or shifts due to interactions with rGO. Importantly, the main MOF-808 diffraction peaks are retained in the MOF-808@rGO composite, indicating that the framework remains structurally intact after hybridization. The coexistence of MOF-808 and rGO features confirms successful composite formation. Compared to pristine MOF-808, the composite shows partial peak broadening, suggesting a degree of amorphization, strong interfacial interaction, or reduced particle size. These structural modifications, including possible changes in pore characteristics, may contribute to enhanced adsorption performance by facilitating improved interaction between the adsorbent and urea molecules.

Fourier Transform Infrared (FT-IR) spectroscopy was used to identify functional groups through characteristic vibrational modes of chemical bonds. The FT-IR spectrum of MOF-808 (Fig. 2b) shows peaks corresponding to the organic linker (benzene tricarboxylic acid) and zirconium clusters, confirming successful framework formation. The MOF-808@rGO composite exhibits similar features to MOF-808, along with additional bands around ~ 1700 cm^−1^ and ~ 3400 cm^−1^. These peaks are associated with oxygen-containing functional groups and hydroxyl vibrations, indicating the presence of residual groups from GO. The broad O–H band suggests hydrogen bonding and/or adsorbed water molecules. The coexistence of these features indicates that the composite retains the structural characteristics of MOF-808 while incorporating rGO. Shifts in peak position and changes in intensity, particularly in the carboxylate and hydroxyl regions, suggest interactions between MOF-808 and rGO. Compared to pure GO (Fig. 2b), the FT-IR spectrum of the composite shows the disappearance of the peak at ~ 1059 cm^−1^, attributed to C–O stretching vibrations of epoxy/hydroxyl groups. This removal is a direct indicator of the deoxygenation of the GO sheets. This chemical reduction is attributed to the synthetic environment, specifically the use of formic acid as a modulator, which also acts as an effective reducing agent under the employed solvothermal conditions (130 °C for 48 h)^58^. The combined action of formic acid and prolonged heating facilitates the cleavage and removal of these oxygen-containing functional groups.

**Fig. 3:**
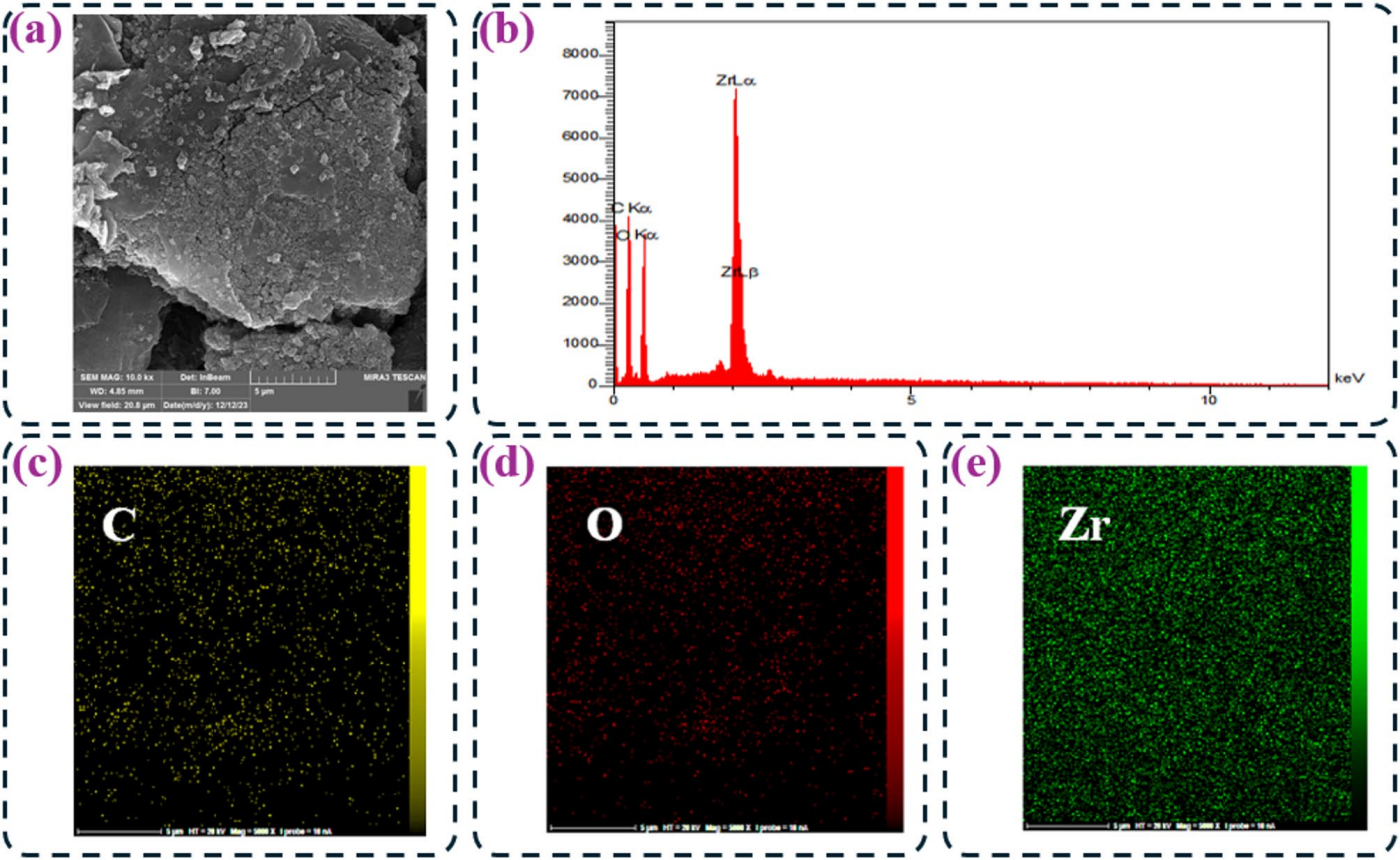
EDS-mapping of MOF-808@rGO: (**a**) SEM image, (**b**) EDS spectrum, and elemental mapping of (**c**) Carbon, (**d**) Oxygen, and (**e**) Zirconium.

Figure 3a presents a SEM image of the MOF-808@rGO composite, indicating the presence of MOF matrix incorporated onto the graphene oxide sheets, which suggests an engineered surface area, robust interaction, and hybridization. The layered structure likely enhances urea trapping by providing increased contact sites. Figure 3b illustrates the EDS Spectrum – elemental composition, which displays the energy-dispersive X-ray spectroscopy (EDS) spectrum that identifies the elements present. The detected Carbon elements come from both the organic linker in MOF-808 and the graphene oxide. The base material is graphene oxide, and the uniform distribution shown in Fig. 3c suggests that MOF-808 is grown in-situ on its surface across the composite. The detected Oxygen elements in Fig. 3d are found in both MOF-808 and rGO functional groups, signifying the presence of functional groups from both rGO and MOF-808, which are crucial for adsorption and maintaining structural integrity. The detected Zirconium elements confirm the presence of the metal cluster in MOF-808, indicating that the MOF-808 particles are well-dispersed and embedded within the carbon matrix as shown in Fig. 3e. The elemental profile confirms the successful synthesis of the composite. The coexistence of Zr, C, and O validates the integration of MOF-808 with graphene oxide.

**Fig. 4:**
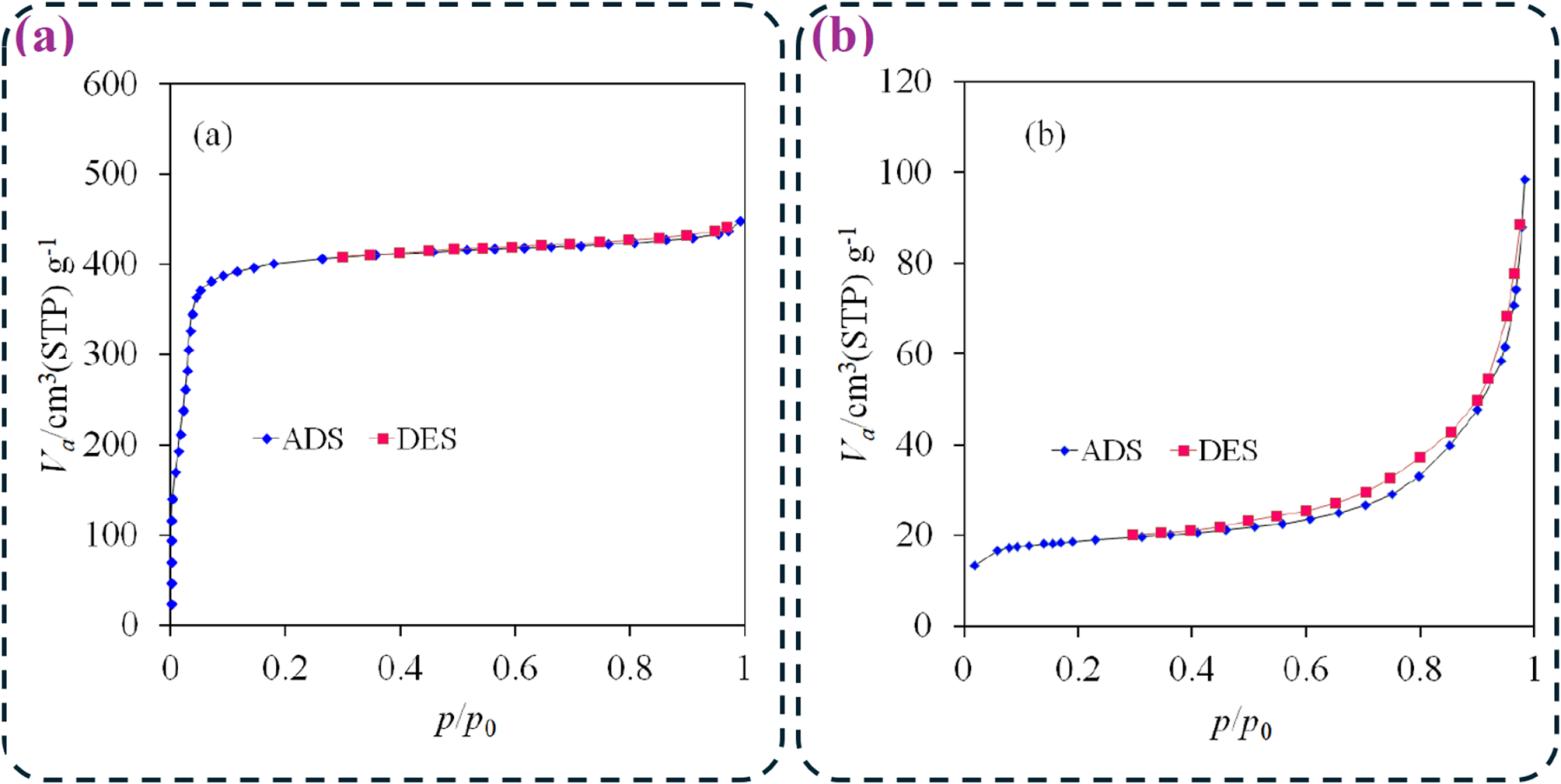
(**a**) N_2_ adsorption and desorption isotherms for (**a**) pure MOF-808 and (**b**) MOF-808@rGO.

**Table 1:** Physical characteristics of the adsorbents determined from adsorption-desorption isotherms of N_2_ gas.

Adsorbent	a_s, BET_ (m^2^/g)	a_micro_ (m^2^/g)	a_ext_ (m^2^/g)	Total pore volume (*p*/*p*_0_ = 0.983) (cm^3^/g)	V_micro_ (cm^3^/g)	Average pore diameter (nm)
MOF-808	1793.5	1042.4	751	0.6914	0.642	1.5421
MOF-808@rGO	74.14	3.072	71.068	0.1524	0.0158	8.223

Figure 4 presents the BET adsorption isotherms of the synthesized composite in comparison with MOF-808, illustrating modifications in pore structure and surface area, including a decrease in pore volume and the emergence of mesoporous porosity following the MOF-808@rGO composite formation. The Table 1 outlines the related data.

The N_2_ adsorption–desorption isotherms of MOF-808 and MOF-808@rGO are presented in Fig. 4a,b. These isotherms were analyzed using the BET theory to evaluate the specific surface area and pore characteristics of the materials. Here, the adsorption capacity refers specifically to the amount of nitrogen adsorbed as a function of relative pressure (p/p_0_)^59^. The isotherm in **graph 4-a** exhibits a steep uptake at low relative pressures (p/p_0_), followed by a plateau as higher pressures. This indicates that the MOF has a high surface area with well-defined microporous structures, enabling strong adsorbate-adsorbent interactions. The rapid initial uptake reflects strong interactions between N_2_ molecules and the microporous framework, while the plateau suggests that most adsorption sites within the MOF are occupied, and adsorption reaches equilibrium.

In contrast, the MOF-808@rGO composite (**graph 4-b**) displays a more gradual adsorption profile without a clear plateau, along with increased uptake at intermediate relative pressures. The reduced N_2_ uptake during in-situ growth of MOF-808 on graphene oxide compared to pure MOF-808 confirms a decrease in accessible surface area and micropore volume upon composite formation and limit nitrogen accessibility during BET analysis. These observations are consistent with the BET results (Table 1), where a significant decrease in specific surface area and micropore volume is observed for the composite. Overall, the BET analysis demonstrates that although the composite exhibits lower nitrogen adsorption capacity compared to pristine MOF-808, the structural modifications play a critical role in tailoring pore architecture and enhancing functional adsorption performance.

**Fig. 5:**
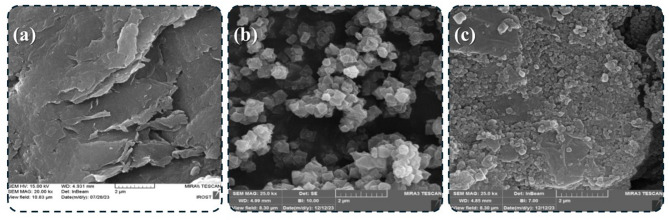
(**a**) SEM images of rGO sheets, displaying a layered, crumpled, and wrinkled morphology with irregular stacking. (**b**) MOF-808 crystals, showing uniformly sized polyhedral structures, which reflect high crystallinity and porosity. (**c**) the MOF-808@rGO composite, showing rGO integrated with MOF particles to form a hierarchical porous network.

**Fig. 6:**
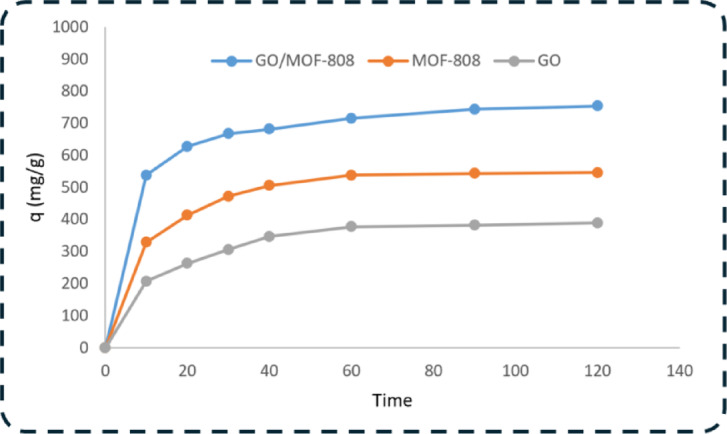
Adsorption performance of MOF-808@rGO composite compared to graphene oxide and MOF individually.

Figure 5 also shows the SEM images of the prepared composite compared to graphene oxide and MOF-808, in which the dispersion of MOF on the graphene surface and the reduction in pore size are clearly evident. The SEM image in Fig. 5-a reveals the characteristic layered and sheet-like morphology of graphene oxide. The sheets appear crumpled and wrinkled, with irregular stacking that contributes to a rough surface texture. These structural features increase the available surface area and provide active sites for adsorption, while also contributing to mechanical flexibility. The presence of wrinkles and folds further enhances adsorption by promoting molecular interactions. The SEM image of MOF-808 (Fig. 5b) shows uniformly distributed polyhedral crystalline particles with well-defined morphology, indicating successful synthesis. Such structures are associated with high porosity and organized pore networks, which are advantageous for the selective adsorption of molecules such as urea. The composite (Fig. 5c) exhibits a hybrid morphology, highlighting the integration of polyhedral MOF structures with crumpled graphene layers, suggesting strong interfacial contact between the two components. This combination leverages the high surface area and mechanical properties of graphene with the intrinsic porosity of MOF-808, potentially enhancing adsorption performance. Furthermore, graphene-based materials typically possess larger and less uniform pores compared to the well-defined micropores of MOFs. In MOF-808@rGO, the coexistence of these features results in a hierarchical pore structure, which may improve adsorption by providing multi-scale pathways for mass transfer. Figure 6 compares the adsorption performance of the MOF-808@rGO composite with that of graphene and MOF-808 individually. The results clearly indicate that the synthesized composite outperforms both graphene and MOF-808, confirming the beneficial synergistic effect of the composite structure.

**Fig. 7:**
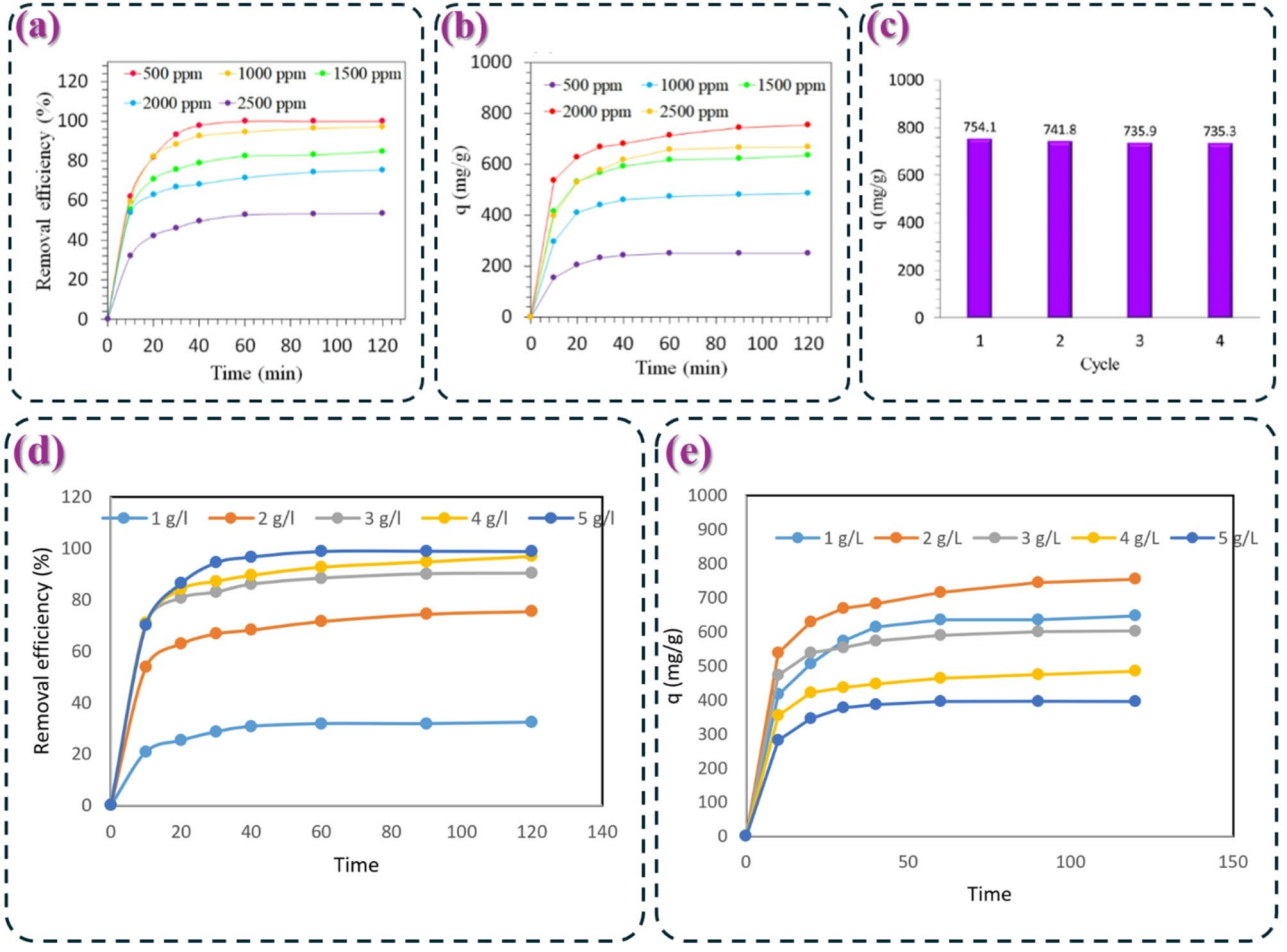
(**a**) Urea removal efficiency of MOF-808@rGO over time at different concentrations. (**b**) Adsorbed urea amounts showing rapid initial uptake and plateauing after ~ 40 min. (**c**) Recyclability of MOF-808@rGO over multiple adsorption cycles. Maximum adsorption is possible in the first cycle because all of the active sites are accessible. However, partial saturation or fouling leads the capacity to drop in later cycles. (**d**) Effect of adsorbent dosage on urea removal efficiency. (**e**) Effect of adsorbent dosage on urea adsorption capacity.

To evaluate the adsorption performance, a certain amount of adsorbent was exposed to aqueous urea solutions with varying initial concentrations, and the change in urea concentration over time was monitored using UV analysis. The results show that MOF-808@rGO achieves complete (100%) urea removal at concentrations below 1000 ppm. The maximum adsorption capacity was approximately 755 mg/g at an initial concentration of 2000 ppm. To determine the recyclability of the adsorbent after urea adsorption, the adsorbent was dispersed in distilled water for 24 h and then separated by centrifugation. After drying at 120 °C, the adsorbent was reused again for urea adsorption. This procedure was repeated for multiple cycles, and the adsorption efficiency was measured after each cycle. The results indicate that the adsorption performance remains stable in an aqueous environment, with only ~ 2.5% reduction in capacity after five cycles. The most significant decrease occurred after the first cycle, while subsequent cycles showed nearly constant performance. This minimal loss demonstrates the good recyclability and reusability of the adsorbent over at least five consecutive cycles. Figure 7 shows the urea adsorption performance of the adsorbent during 5 consecutive periods.

In Fig. 7a, the removal efficiency increases rapidly initially and then reaches a plateau for all urea concentrations. At 500 ppm, the removal efficiency reaches nearly 100% within 40 min and remains constant thereafter. The efficiency levels off at roughly 80% at 1500 ppm, whereas at 2500 ppm it reaches its lowest value, stabilizing at about 60%. The removal efficiency decreases with increasing urea concentration, indicating saturation of the adsorption sites on the MOF-808@rGO composite. At higher concentrations, the number of urea molecules exceeds the available adsorption sites on the MOF-808@rGO composite. This leads to a lower percentage of urea being adsorbed relative to the total amount present^60,61^. Saturation of adsorption sites occurs more quickly at higher concentrations, thereby limiting the removal efficiency. This behavior suggests that the interaction energy between urea molecules and adsorption sites decreases as the surface becomes saturated^61,62^. In Fig. 7b, the adsorption performance increases rapidly initially and then levels off, similar to the trend observed for removal efficiency. The trends stabilize after 40 min. The adsorption performance reaches a maximum of 800 mg/g at 2500 ppm, while at 500 ppm it stabilizes at about 200 mg/g. In contrast to removal efficiency, adsorption performance increases with increasing urea concentration. This increase is attributed to the greater availability of urea molecules to interact with the adsorption sites at higher concentrations. Even though the removal efficiency decreases, the absolute quantity of urea adsorbed rises due to the higher initial concentration of urea molecules^61,63^. Previous studies on the MOF-808@rGO composite have demonstrated that its engineered surface area and porosity of these materials enable increased adsorption capacity at higher concentrations^62–66^.

**Fig. 8:**
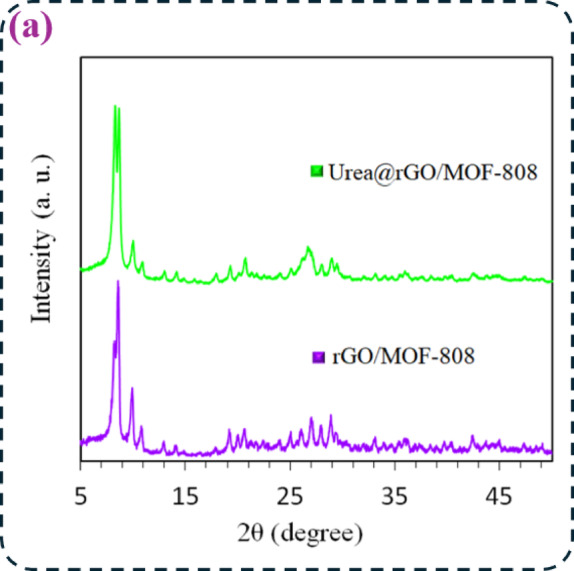
PXRD patterns illustrate the structural stability of the MOF-808@rGO composite before and after urea adsorption. This confirms the robustness and reusability of the composite material for urea removal applications.

In Fig. 7c, the bar graph illustrates the urea adsorption capacity [mg/g] over four sequential recycling cycles of the MOF-808@rGO composite. During the first cycle, the active sites on the MOF-808@rGO composite are fully available for urea adsorption. However, subsequent cycles may lead to partial saturation or fouling of these sites by residual urea molecules or impurities, thereby reducing adsorption capacity and chemical interactions. Repeated cycles can also alter the structural integrity of the MOF-808@rGO composite, leading to pore collapse or reduced surface area. These changes diminish the accessibility of active sites for urea molecules^62,67,68^. In addition, in Fig. 8 presents PXRD analysis used to evaluate the stability of the MOF-808@rGO composite structure after 5 adsorption–desorption cycles. The X-ray diffraction pattern confirms that the composite retains its structure stability after urea adsorption.

PXRD was employed to examine the crystalline structure of the MOF-808@rGO composite before and after urea adsorption. The diffraction patterns were obtained by measuring the intensity of diffracted X-rays as a function of the diffraction angle (2θ)^69–71^. As shown in Fig. 8, the PXRD pattern exhibits well-defined diffraction peaks, indicating a high degree of crystallinity. The observed peak positions are consistent with the characteristic reflections of MOF-808, while broad features associated with graphene oxide are also present, confirming the successful composite formation^69–71^. After urea adsorption, the main diffraction peaks remain at similar positions, demonstrating that the overall crystalline framework of MOF-808@rGO is preserved during the adsorption process^69,72^. Minor variations in peak intensity are observed, which may arise from changes in crystallite orientation, surface coverage, or adsorption-induced effects. However, these variations do not indicate any phase change or framework collapse^73,74^. Overall, the PXRD results confirm the structural stability of the MOF-808@rGO composite upon urea adsorption, supporting its suitability for repeated adsorption applications^69–71^.

**Fig. 9:**
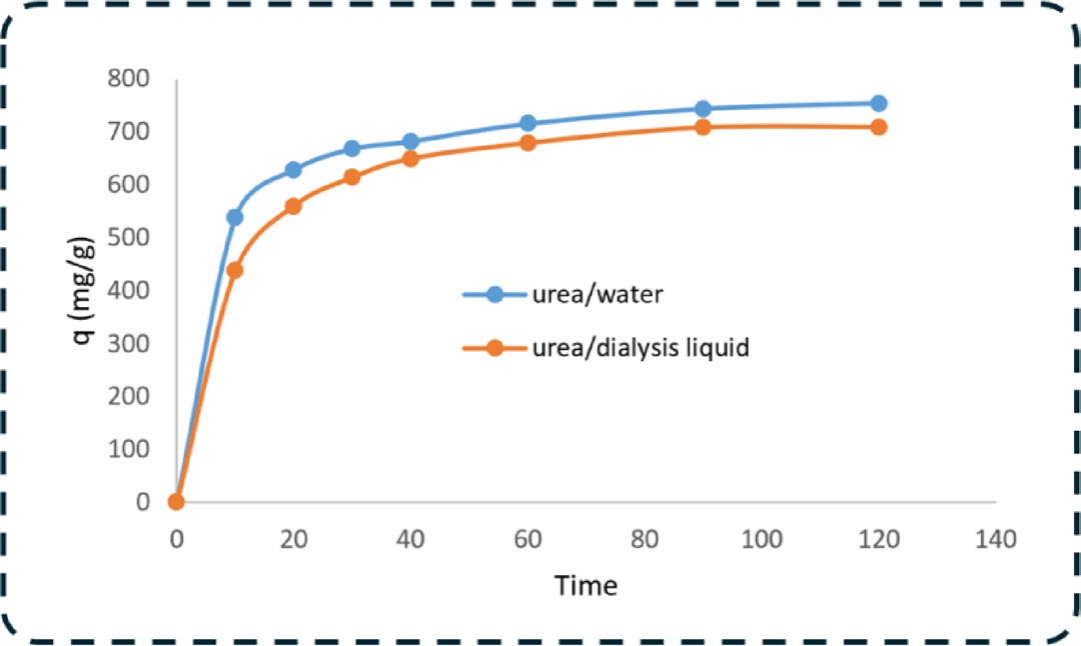
comparative adsorption performance of MOF-808@rGO for urea removal in aqueous and dialysis solutions.

Figure 9 illustrates the comparative adsorption performance of MOF-808@rGO composite for urea removal from both aqueous and dialysis solutions. As observed, the adsorption capacity shows a slight decrease in the dialysis solution compared to the aqueous medium. This reduction is attributed to the presence of ions and other chemical species in the dialysis solution, which compete with urea molecules for active adsorption sites. These results indicate that the MOF-808@rGO composite exhibits considerable adsorption capacity toward urea, even in the presence of competing ions in the dialysis solution. This finding confirms its potential for application in wearable dialysis systems.

### Adsorption kinetics and isotherm modeling

To evaluate the adsorption kinetics and identify the rate-limiting mechanisms, the pseudo-first-order, pseudo-second-order, and intra-particle diffusion kinetic models were applied. The linearized expressions of these models are presented in Eqs. (1–3), respectively. These models are widely employed for fitting experimental adsorption kinetic data:

1 $$\mathrm{n}\left({\mathrm{q}}_{\mathrm{e}}-{\mathrm{q}}_{\mathrm{t}}\right)=\mathrm{ln}\left({\mathrm{q}}_{\mathrm{e}}\right)-{\mathrm{k}}_{1}\mathrm{t}$$

2 $$\frac{\mathrm{t}}{{\mathrm{q}}_{\mathrm{t}}}=\frac{1}{{\mathrm{k}}_{2}{\mathrm{q}}_{\mathrm{e}}^{2}}+\frac{\mathrm{t}}{{\mathrm{q}}_{\mathrm{e}}}$$

3 $$\:{\mathrm{q}}_{\mathrm{t}}={\mathrm{k}}_{\mathrm{i}\mathrm{d}}{\mathrm{t}}^{0.5}+\mathrm{C}$$

where $\:t$ denotes the contact time (min). The constants $\:{k}_{1}$ (min^−1^), $\:{k}_{2}$ (g /mg. min), and $\:{k}_{id}$ (mg/g. min^0.5^) correspond to the rate constants of the pseudo-first-order, pseudo-second-order, and intra-particle diffusion models, respectively. The intercept $\:C$ (mg/g) is associated with the boundary layer thickness and reflects the contribution of external mass transfer resistance.

**Fig. 10:**
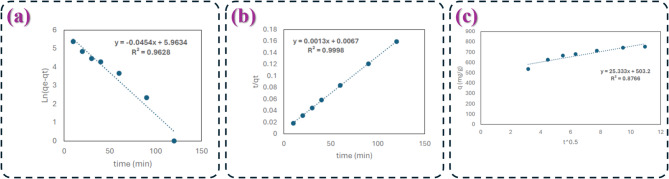
(**a**) Kinetic models for urea adsorption on MOF-808@rGO (**a**) Pseudo-first-order kinetic model (**b**) Pseudo-second-order kinetic model (**c**) Intra-particle diffusion kinetic model.

Based on the results presented in Fig. 10; Table 2, the pseudo-second-order kinetic model provides a noticeably better fit to the experimental data than the pseudo-first-order and intra-particle diffusion models. This observation indicates that urea adsorption is mainly governed by a pseudo-second-order mechanism, suggesting that chemisorption is the dominant process controlling the interaction between urea molecules and the adsorbent surface^75^.

**Table 2:** The kinetic factors associated with urea adsorption on MOF-808@rGO.

Pseudo-first-order			Pseudo-second-order			Intra-particle diffusion		
q_e_ (mg/g)	K_1_ (min^−1^)	R^2^	q_e_ (mg/g)	K_2_ (g/mg.min)	R^2^	C (mg/g)	K_id_ (mg/g.min^0.5^)	R^2^
388.9302	0.0454	0.9628	769.2308	0.000252	0.9998	503.2	25.333	0.8766

To investigate the adsorption mechanism and equilibrium behavior, the Langmuir, Freundlich, and Temkin isotherm models were applied. The linearized forms of these models are given in Eqs. (4)–(6), respectively:

4 $$\:\frac{{\mathrm{C}}_{\mathrm{e}}}{{\mathrm{q}}_{\mathrm{e}}}=\frac{1}{{\mathrm{K}}_{\mathrm{L}}{\mathrm{q}}_{\mathrm{m}\mathrm{a}\mathrm{x}}}+\frac{{\mathrm{C}}_{\mathrm{e}}}{{\mathrm{q}}_{\mathrm{m}\mathrm{a}\mathrm{x}}}$$

5 $$\mathrm{l}\mathrm{n}{\:\mathrm{q}}_{\mathrm{e}}=\mathrm{l}\mathrm{n}{\mathrm{K}}_{\mathrm{F}}+\frac{1}{{\mathrm{n}}_{\mathrm{F}}}\mathrm{l}\mathrm{n}{\:\mathrm{C}}_{\mathrm{e}}$$

6 $${\mathrm{q}}_{\mathrm{e}}=\frac{\mathrm{R}\mathrm{T}}{{\mathrm{B}}_{\mathrm{T}}}\:\mathrm{l}\mathrm{n}{\mathrm{K}}_{\mathrm{T}}+\frac{\mathrm{R}\mathrm{T}}{{\mathrm{B}}_{\mathrm{T}}}\mathrm{ln}{\mathrm{C}}_{\mathrm{e}}$$

In the Langmuir model, $\:{\mathrm{q}}_{\mathrm{m}\mathrm{a}\mathrm{x}}$ (mg/g) represents the maximum monolayer adsorption capacity, while $\:{\mathrm{K}}_{\mathrm{L}}$ (L/mg) is the Langmuir constant related to the affinity between the adsorbate and the adsorption sites. For the Freundlich model, $\:{\mathrm{K}}_{\mathrm{F}}$ (mg/g) is associated with adsorption capacity, and $\:{\mathrm{n}}_{\mathrm{F}}$ indicates adsorption intensity. In the Temkin model, $\:\mathrm{T}$ (K) is the absolute temperature, $\:\mathrm{R}$ (8.314 J/mol.K) is the gas constant, $\:{\mathrm{K}}_{\mathrm{T}}$ (L/mol) represents the equilibrium binding constant, and $\:{\mathrm{B}}_{\mathrm{T}}$ (J/mol) is related to the heat of adsorption.

**Fig. 11:**
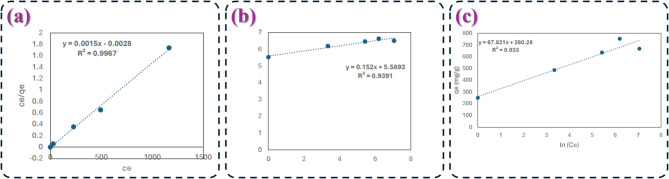
Adsorption isotherm of urea on the MOF-808@rGO adsorbent (**a**) Langmuir (**b**) Freundlich (**c**) Temkin.

Based on the experimental data (Fig. 11; Table 3), the Langmuir isotherm model exhibited the best fit among the studied models. This indicates that urea adsorption occurs primarily as monolayer coverage on a homogeneous surface with a finite number of identical adsorption sites^76^.

**Table 3:** Calculated isotherm parameters for adsorption of urea on MOF-808@rGO.

Langmuir			Freundlich			Temkin		
q_max_ (mg/g)	K_L_ (L/mg)	R^2^	n_F_	K_F_ (mg/g)	R^2^	B_T_ (J/mol)	K_T_ (L/mol)	R^2^
666.6667	0.5357	0.9967	6.5789	267.5483	0.9391	36.544	46.3946	0.933

**Fig. 12:**
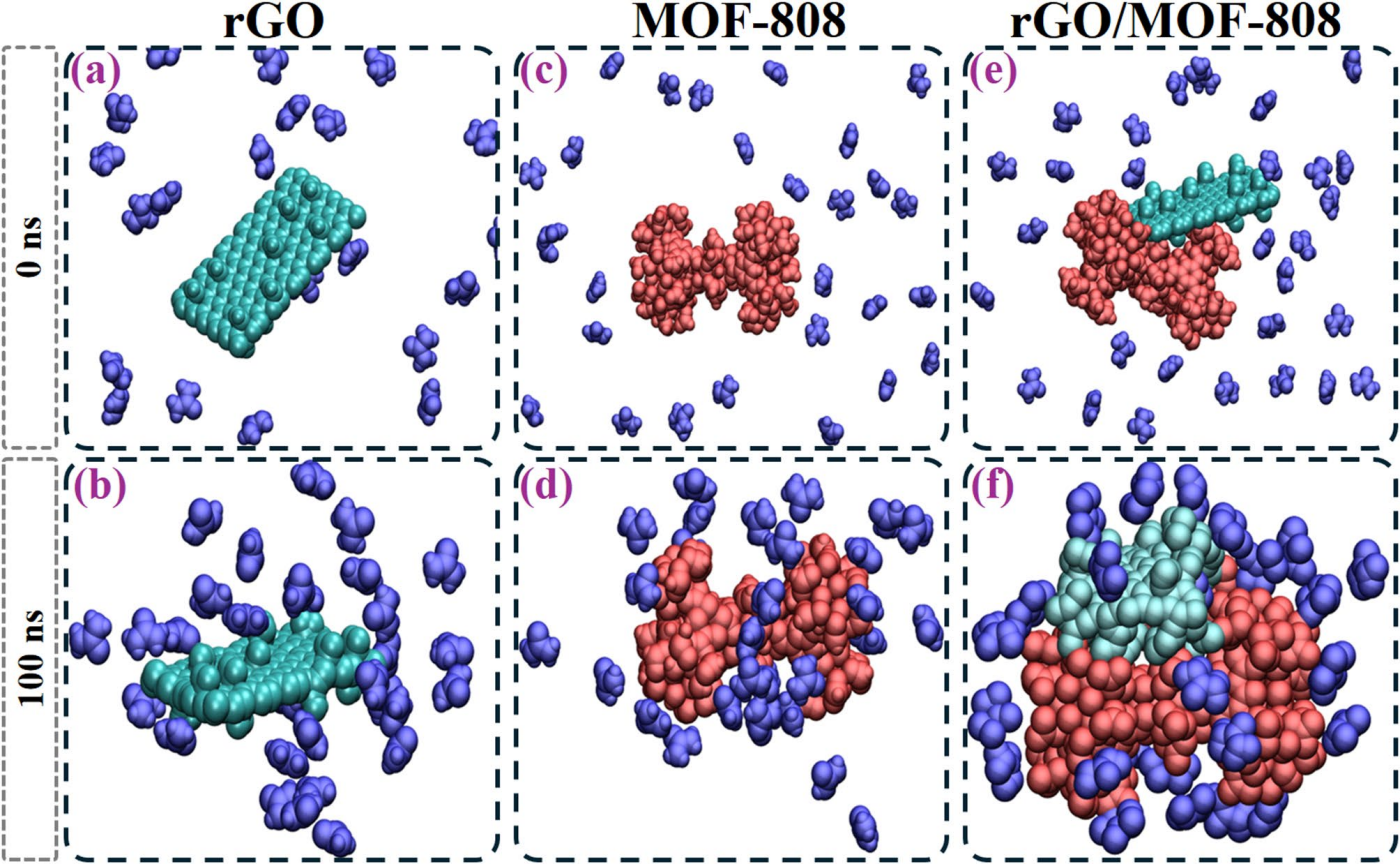
Molecular simulations showing (**a**, **b**) 3D model evolution of GO functional groups during urea adsorption process, (**c**, **d**) structural configurations of pure MOF-808 and MOF-808 in solution at initial (0 ns) and final (100 ns) simulation stages, and (**e**, **f**) MOF-808@rGO composite interactions with urea over time, highlighting hydrogen bonding and coordination interactions.

Graphene oxide (GO) is characterized by the presence of oxygen-containing functional groups such as hydroxyl (–OH), epoxy (–O–), and carboxyl (–COOH). This functionalization provides active sites for further chemical interactions. The sp^2^-hybridized carbon lattice of graphene allows the incorporation of oxygen-containing functional groups through oxidative processes^77^. These processes break some of the aromatic π-bonds, creating reactive sites where oxygen atoms or functional groups can covalently bond^78^. Upon oxidation, hydroxyl and carboxyl groups on graphene oxide can form hydrogen bonds with water or other molecules, thereby influencing dispersion and stability in liquid environments. In addition, non-covalent van der Waals interaction between the graphene oxide layers and external molecules can impact aggregation and interaction dynamics. Furthermore, the presence of charged functional groups enables electrostatic interactions with other species^77–79^. In Fig. 12a,b, the 3D model illustrates how these functional groups and bonding mechanisms evolve during simulations. Over time, the incorporation of oxygen-containing functional groups modifies the surface chemistry and physical properties of graphene, enhancing its suitability for urea removal.

MOF-808 is a fascinating metal-organic framework formed through the coordination of zirconium (Zr) clusters with organic linkers, typically benzene-1,3,5-tricarboxylate (BTC). The molecular structure generally consists of Zr_6_O_4_(OH)_4_ clusters, where zirconium atoms are bonded to oxygen atoms, while BTC molecules act as linkers that connect the zirconium clusters into a stable and porous network^80^. Thus, Zirconium atoms in Zr_6_ clusters form coordination bonds with the carboxylate groups of the BTC linkers. These bonds are the backbone of the MOF structure^81,82^. Hydroxyl groups (–OH) in the Zr clusters, as well as uncoordinated carboxyl groups in the framework, can form hydrogen bonds with surrounding molecules during adsorption or interaction. Van der Waals interactions occur between the framework and guest molecules such as urea, playing a role in stabilizing adsorbed species^83^. When graphene is incorporated, its delocalized π-electron system can interact with the aromatic rings of the BTC linkers^84,85^.

As shown in Fig. 12c,d, the molecular configuration evolves over time due to interactions between MOF-808 and surrounding molecules. These interactions provide insight into the mechanism of urea removal, as the porous framework enables adsorption and potential chemical reactions. Figure 12e,f illustrates the molecular evolution of MOF-808@rGO in the presence of urea over time, from 0ns to 100ns. At 0ns, MOF-808 (red) and graphene oxide (cyan) are dispersed, whereas urea molecules (blue) are distributed throughout the medium. The molecular structure appears loosely arranged, indicating minimal interaction between the components at this initial stage. At 100ns, the MOF-808@rGO structure becomes more compact and integrated^67^. Urea molecules become embedded within the composite, suggesting significant interaction and bonding over time^41^. In this process, urea molecules form hydrogen bonds with oxygen-containing functional groups in graphene oxide and the metal centers in MOF-808. The metal centers in MOF-808 coordinate with oxygen atoms in graphene oxide, enhancing the stability of the composite^67^. Van der Waals forces facilitate the initial attraction and alignment between MOF-808 and graphene oxide^67^.

The material’s capability to remove urea is improved by hydrogen and coordination bonds, which strengthen the interactions and create a stable composite structure^86^. This indicates that strong bonding between MOF-808 and graphene oxide ensures the durability and effectiveness of the composite. This effectively traps urea molecules inside the composite^67^.

**Fig. 13:**
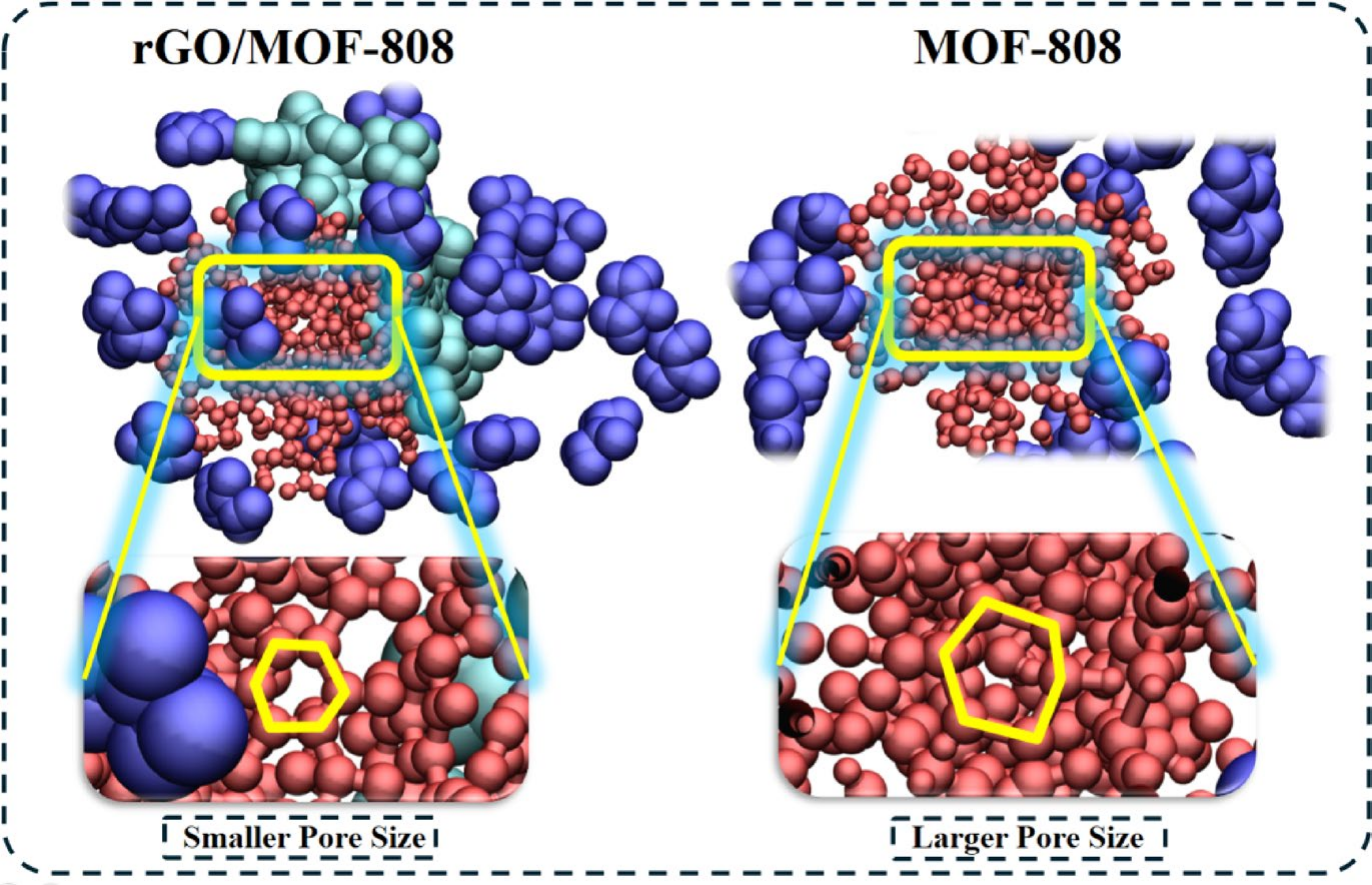
Pore size distribution of pure MOF-808 and MOF-808@rGO, resulting in a reduction of pore sizes due to rGO sheets occupying space within the framework and increased active site density due to rGO incorporation, enhancing urea adsorption.

Figure 13 compares the pore sizes of pure MOF-808 and MOF-808@rGO composite. On the left, the molecular configuration of MOF-808 with GO shows the modified pore sizes, while the right side illustrates the larger pore sizes of pure MOF-808. During composite formation, MOF-808 is incorporated onto the rGO sheets, which leads to a reduction in pore size as the GO sheets occupy space within the MOF framework^87^. The oxygen-containing functional groups in GO, such as hydroxyl and carboxyl groups, interact with the metal nodes and organic linkers of MOF-808, altering their structural arrangement^38^. This modification reduces the available pore volume while increasing the density of active sites, thereby enhancing the adsorption efficiency for urea molecules. The modified pores structure provides increased surface area and stronger interactions with urea, making the composite highly effective for urea removal^38,87,88^.

The relationship between pore size and adsorption efficiency depends on balancing diffusion and adsorbate–adsorbent interactions. Excessively small pores can restrict mass transfer and reduce accessibility, whereas moderate pore confinement enhances adsorption by strengthening molecular interactions. In MOF-808@rGO, pore size decreases while still allowing access for small urea molecules. This controlled confinement intensifies host–guest interactions within the composite. Consequently, urea is retained more effectively inside the pores, improving overall adsorption performance. Experimental and atomistic observations support these findings, showing stronger interactions and retention without notable diffusion limitations. This indicates an optimal structural balance that maximizes adsorption capacity and maintains efficient transport.

Table 4 compiles the experimental conditions and urea adsorption data from previously published studies employing organic and inorganic adsorbents. The inclusion of the present work in the last row enables a direct comparison and highlights the contribution and performance of the current study in relation to earlier reports.

**Table 4:** Application of various materials as organic/inorganic adsorbents and their results in urea adsorption.

Type of material	Adsorption conditions	Urea removal (%)	Reference
2D materials (MoO_3_-COOH)	Computational simulation	91.75% of removal	^1^
Graphene oxide (GO)	0.4 g adsorbent, 200mL of a simulated serum solution at 37 °C	0.45 mmol/gr	^89^
Modified Graphene after acid treatment	50 mg adsorbent, 20 mL of 160 mg/L, 37 °C at 4 h	75% of removal	^90^
Graphene based Nylon-6/GNP/CB 0.25%	in a time of 3 h with 10 min after simulating a dialysis for 4 h.	90% of removal	^91^
Modified Graphene with amine mixtures	50 mg adsorbent, 20 mL of 160 mg/L, 37 °C at 4 h	97% of removal	^92^
MXene	5 g adsorbent, 6 mL of 300 mg/L urea solution,, 37 °C	94% of removal	^93^
N8-doped Fullerene	Computational simulation	Qualitative description	^94^
Organic [COS/P(AM-co-SSS)] monolith	20 mg adsorbent, 20 mL of 100 mg/L urea solution at 25 °C	199.3 mg/g for urea	^95^
MOF with Cu-BTC nanofiber membranes	52.2 mg adsorbent, 150 mL of 100 mg/L urea solution at 37 °C	92.8% of removal	^96^
Isoreticular MOFs @SiO_2_	10 mg adsorbent, 30 mL of 100 mg/L urea solution at 37 °C	92.57% of removal	^97^
AMMT-HMOF	1 mL of 20 mg/L of solution, within 1 h after recycling 4 times.	80% of removal	^98^
Bio-MOF-11 (YUVSUE)	Computational simulation at 37 °C and 1 bar	Max adsorbed amount of urea: 38.68 mg/g	^99^
Bio-MOF-12 (BEYSEF)	Computational simulation at 37 °C and 1 bar	Max adsorbed amount of urea: 63.64 mg/g	^99^
Bio-MOF (OREZES)	Computational simulation at infinite dilution and 37 °C	Max membrane selectivity: 347.94 for urea/water	^100^
Fe-based MOFs (MIL-100(Fe))	25–400 ppm MOF and 25–1000 ppm urea, pH (2–10) at 25–65 °C	86% removal within 2 min	^101^
Cu-based MOFs (Cu_3_(BTC)_2_)	50 ml of 0.1 M urea solution at 50 °C	Max guest loading: 30 wt% of urea	^102^
Zr-MOF/rGO composite	5 g/L adsorbent, 100 mL of 2000 ppm urea solution	100% of removal	This Work

## Conclusion and future perspective

This paper offers an in-depth analysis of the synthesis and utilization of the MOF-808@rGO composite for efficient urea removal, targeting artificial kidney technologies. The development of MOF–graphene composites reflect a meaningful progress in materials science, especially in addressing urea accumulation in patients with compromised renal function. Our findings demonstrate that the MOF-808@rGO composites exhibit remarkable stability and modified pore accessibility, which are critical attributes for enhancing urea adsorption capabilities.

Initially, we synthesized the MOF-808@rGO composite through a well-structured experimental process, involving the dispersion of graphene oxide in a suitable solvent followed by necessary thermal treatments. Structural characterizations (XRD, FT-IR, SEM, BET, and EDS) confirmed the successful composite formation of MOF-808@rGO, resulting in a hybrid material with a hierarchical pore structure, enhanced interfacial interactions, and preserved crystallinity. The surface properties and porosity of the MOF not only enhance the adsorption capacity but also facilitate the effective trapping of urea molecules, addressing one of the critical challenges in urea management. These structural changes improved urea confinement and strengthened adsorbate–adsorbent interactions, which was further supported by molecular simulations showing hydrogen bonding and coordination interactions between urea molecules and the composite framework.

Experimentally, the MOF-808@rGO composite exhibited excellent adsorption performance, achieving a maximum adsorption capacity of approximately 755 mg/g and complete urea removal under optimized conditions (2000 ppm, 5 g/L adsorbent dosage), highlighting the potential of MOF-808@rGO as an advanced material for renal treatment technologies. The adsorption process followed a pseudo-second-order kinetic model, indicating that chemisorption dominates the mechanism, while equilibrium data were best described by the Langmuir isotherm, suggesting monolayer adsorption on homogeneous active sites. In addition to high efficiency, the composite demonstrated strong stability and reusability, with only a minor (~ 2.5%) reduction in adsorption capacity after five cycles. The material also maintained considerable performance in dialysis-like environments despite the presence of competing ions, highlighting its practical applicability [3,68,103].

Furthermore, the study delves into the interaction mechanisms at an atomic level through atomistic simulations. The results reveal that the in-situ growth of MOF-808 on graphene oxide leads to a reduction in pore size, which subsequently enhances the entrapment efficiency of urea molecules. This unique interplay between the porous nature of the MOFs and the presence of graphene oxide generates a synergistic effect that enhances the overall efficiency of urea removal processes compared to traditional methods. This work underscores the importance of pore engineering and hybrid material design in enhancing adsorption performance and presents MOF-808@rGO as a promising candidate for urea removal in advanced water treatment and wearable dialysis systems. Future studies should address the scalability and durability of these materials under varying conditions. Improved understanding of atomistic interactions will enable the design of targeted adsorbents for specific contaminants. This work lays the foundation for next-generation materials in kidney disease management and may contribute to redefining hemodialysis strategies. Ongoing exploration of emerging structures further underscores the potential of MOF-808@rGO as a promising platform for artificial kidney technologies.

## Data Availability

The datasets analyzed/generated during the current study available from the corresponding author on reasonable request.
